# 1986. Switching to DOR/3TC/TDF Improves Renal Function and Decreases Lipid Levels in People Living With HIV: a Real-Life Multicentric Study

**DOI:** 10.1093/ofid/ofad500.113

**Published:** 2023-11-27

**Authors:** Cristina Micali, Ylenia Russotto, Medical Doctor, Giovanni Francesco Pellicanò, Letizia Panella, Antonio Albanese, Laura Santoro, Sonia Agata Sofia, Grazia Pantò, Mariagiovanna Coco, Alessandro Guarneri, Carmelo Iacobello, Michele Salvatore Paternò Raddusa, Manuela Ceccarelli, Andrea Marino, Benedetto Maurizio Celesia, Bruno Santi Cacopardo, Arturo Montineri, Chiara Frasca, Agnese Colpani, Andrea De Vito, Giordano Madeddu, Angela Alibrandi, Emmanuele Venanzi Rullo, Giuseppe Nunnari

**Affiliations:** University of Messina, Messina, Sicilia, Italy; Unit of Infectious Diseases, Department of Clinical and Experimental Medicine, University of Messina, 98124 Messina, Italy, Messina, Sicilia, Italy; Unit of Infectious Diseases, Department of Adult and Childhood Human Pathology “Gaetano Barresi”, University of Messina, 98124 Messina, Italy, Messina, Sicilia, Italy; Cutroni-Zodda Hospital, Messina, Sicilia, Italy; Papardo Hospital, Messina, Sicilia, Italy; University of Messina, Messina, Sicilia, Italy; Azienda Ospedaliera per l'Emergenza Cannizzaro, Catania, Sicilia, Italy; Azienda Ospedaliera per l'Emergenza Cannizzaro, Catania, Sicilia, Italy; University of Messina, Messina, Sicilia, Italy; University of Messina, Messina, Sicilia, Italy; University of Catania, Catania, Sicilia, Italy; University of Messina, Messina, Sicilia, Italy; University of Catania, Catania, Sicilia, Italy; University of Catania, Catania, Sicilia, Italy; University of Catania, Catania, Sicilia, Italy; University of Catania, Catania, Sicilia, Italy; G. Rodolico - San Marco Hospital, Catania, Sicilia, Italy; G. Rodolico - San Marco Hospital, Catania, Sicilia, Italy; University of Sassari, Sassari, Sardegna, Italy; University of Sassari, Sassari, Sardegna, Italy; University of Sassari, Sassari, Sardegna, Italy; University of Messina, Messina, Sicilia, Italy; University of Messina, Messina, Sicilia, Italy; Unit of Infectious Diseases, Department of Clinical and Experimental Medicine, University of Catania, 95131 Catania, Italy, Catania, Sicilia, Italy

## Abstract

**Background:**

Pivotal studies on Doravirine (DOR) showed its high tolerability and effectiveness, even on resistant strains. Therefore, DOR and its single tablet combination treatment DOR/lamivudine (3TC)/tenofovir disoproxil fumarate (TDF) has been referred to as a cardiovascular-safe drug. However, data from real-life studies on safety and efficacy of DOR are lacking.

**Methods:**

Retrospective multicenter cohort study, including 6 Italian HIV centers, evaluated adult treatment-experienced People Living With HIV (PLWH) who switched to DOR/3TC/TDF from June 2020 to June 2022. PLWH with known resistance mutations to DOR were excluded. Clinical and laboratory data were collected at the time of the switch and after 48 weeks.

**Results:**

A total of 98 PLWH were switched to a DOR/3TC/TDF regimen, 75 (76.5%) male, with a median age of 52.41 years (IQR 42.95-58.79).

The main reason for switching to DOR/FTC/TDF regimens was proactive switch (33.3%). No differences were found between the two timepoints on plasma viral load (pVL) (p = 0.345), lymphocytes T CD4+ (CD4+) percentage (p = 0.994) and count (p = 0.611), and CD4+/lymphocytes T CD8+ (CD8+) ratio (p = 0.899), confirming the effectiveness of the drug combination. We also found a significant reduction in total (p < 0.001) and LDL (p = 0.025) cholesterol and triglycerides (p = 0.002). Importantly, we found that the estimated glomerular filtration rate (eGFR) calculated with CKD-EPI formula significantly improved despite the presence of TDF (p = 0.028).

Adverse events (AEs) were reported in 2 PLWHs and were characterized by the onset of a nodular rash in both arms and legs after 2 weeks of therapy in one PLWH, and by the onset of a progressive paresthesia in both extremities of arms after one week of treatment in the other PLWH. In both cases the treatment with DOR/3TC/TDF was interrupted.

**Conclusion:**

The present real-life study showed that DOR/3TC/TDF significantly reduced lipid levels, surprisingly improving estimated renal function. The virological efficacy of DOR/3TC/TDF along with its immunological, metabolic and safety profile are key elements for a proactive management of the cardiovascular risk.

**Disclosures:**

**All Authors**: No reported disclosures

